# Prevalence and Associated Factors of Caesarean Section and its Impact on Early Initiation of Breastfeeding in Abu Dhabi, United Arab Emirates

**DOI:** 10.3390/nu11112723

**Published:** 2019-11-10

**Authors:** Zainab Taha, Ahmed Ali Hassan, Ludmilla Wikkeling-Scott, Dimitrios Papandreou

**Affiliations:** 1 Department of Health Sciences, College of Natural and Health Sciences, Zayed University, Abu Dhabi, P.O. Box 144534, UAE; ludmilla.scott@gmail.com (L.W.-S.); Dimitrios.Papandreou@zu.ac.ae (D.P.); 2 Taami for Agricultural and Animal Production, Khartoum, Sudan; aa801181@gmail.com

**Keywords:** caesarean section, initiation of breastfeeding, maternal age, gestational age, United Arab Emirates

## Abstract

The World Health Organization (WHO) recommends the early initiation of breastfeeding. Research shows that factors such as mode of delivery may interfere with the early initiation of breastfeeding. However, data in the United Arab Emirates (UAE) on these findings is limited. Thus, the aim of this study was to describe the prevalence of caesarean sections (CSs) and evaluate their effect on breastfeeding initiation among mothers of children under the age of two years in Abu Dhabi. Data were collected in clinical and non-clinical settings across various geographical areas in Abu Dhabi during 2017 using consent and structured questionnaires for interviews with mothers. Data analysis included both descriptive and inferential statistics. Among the 1624 participants, one-third (30.2%) reportedly delivered by CS, of which 71.1% were planned, while 28.9% were emergency CS. More than half of all mothers (62.5%) initiated early breastfeeding. Multivariable logistic regression indicated factors that were associated positively with CS included advanced maternal age, nationality, and obesity. However, gestational age (GA) was negatively associated with CS. This study shows that the prevalence of CS is high in Abu Dhabi, UAE. CS is associated with lower early initiation rates of breastfeeding. The early initiation rates of breastfeeding were 804 (79.2%) 95% confidence interval (CI) (76.4, 82.0), 162 (16.0%) 95% CI (10.4, 21.6), and 49 (4.8%) 95% CI (1.2, 10.8) among vaginal delivery, planned CS, and emergency CS, respectively. Regarding the mode of delivery, vaginal were 2.78 (Adjusted Odd Ratio (AOR)): CI (95%), (2.17–3.56, *p* < 0.001) times more likely related to an early initiation of breastfeeding. CS in general, and emergency CS, was the main risk factor for the delayed initiation of breastfeeding. The study provides valuable information to develop appropriate strategies to reduce the CS rate in UAE. Maternal literacy on CS choices, the importance of breastfeeding for child health, and additional guidance for mothers and their families are necessary to achieve better breastfeeding outcomes.

## 1. Introduction

The rate of caesarean section (CS) in the United Arab Emirates (UAE) has increased from 10% in 1995 [1] to 24% in 2014 [2]. The World Health Organization (WHO) [3] suggests a CS rate of 10%–15% of all live births, which is significantly lower than those reported in the UAE. Researchers suggest that the mode of delivery influences breastfeeding initiation and duration [4,5], and may influence subsequent breastfeeding and duration [6].

CS is well documented to be associated with suboptimal consequences related to both the mother and her infant’s health [7,8]. Among those consequences reported, endometritis, hemorrhage, cystitis, infant respiratory complications, and hypoglycemia [8,9] may have negative effects on breastfeeding. The effect of CS on the initiation of breastfeeding may be related to the adverse effects of anesthesia for both mothers and their newborns. Maternal distress, which often accompanies CS, may negatively affect the baby’s feeding behaviors and breastfeeding outcomes. 

An abundance of studies have shown that mothers who give birth via CS delivery may be less likely to breastfeed, and or more likely to delay breastfeeding initiation [10,11,12]. An early initiation of breastfeeding, i.e., within the first hour after delivery, has been recommended by the WHO as an important factor to extend breastfeeding duration [13,14,15]. An important practice recommended by the WHO, as part of the 10 steps of the Baby-Friendly Hospital Initiative (BFHI), is skin-to-skin contact [16]. To ensure this practice, the mother and her newborn infant must be conscious and fully awake. Therefore, babies born by CS may not benefit from skin-to-skin contact immediately after birth, and may be more susceptible to delayed breastfeeding [16]. Previous studies have found that delaying breastfeeding initiation that co-occurs with CS delivery is associated with factors such as mother–baby separation, impaired suckling skills, and insufficient milk production. This will ultimately impact the continuation of breastfeeding [10,17,18].

The health benefits of breastfeeding for mother and child have been well documented. Mothers who breastfeed their babies have a reduced incidence of type 2 diabetes mellitus, as well as breast and ovarian cancers [19]. Moreover, breastfed babies are less likely to develop childhood illnesses and obesity, and have higher levels of intelligence as an adult [20,21].

Following the WHO recommendations [14], the UAE has incorporated the Global Strategy for Infant and Young Child Feeding, and the Ministry of Health (MOH) has issued a national infant feeding policy [22]. This policy states that infants should be breast fed exclusively until six months of age and continue breastfeeding up to or beyond two years of age [22]. A previous study by Taha et al. revealed a breastfeeding initiation rate of 95.6% and early initiation of 62.5% [23]. However, only 7% of babies were still breastfeeding at six months of age, which prompted the authors to further investigate these findings.

Multiple studies from different countries on the effects of CS versus vaginal delivery on breastfeeding practices have shown contradicting results [24,25,26,27]. While some research has documented major barriers to breastfeeding after CS [28,29,30], other studies have indicated that CS had no effect on breastfeeding initiation [31,32] and duration [33]. Some studies reported that planned CS was associated with delayed breastfeeding [25,34] while others concluded the opposite [35]. 

Regardless of the discrepancies in previous results, CS will remain a significant and alarming concern, as it influences the initiation of breastfeeding. For a country such as the UAE, which follows the WHO recommendations of infants’ and young children’s feeding, but still encounters increased rates of CS deliveries, further investigation and more implementation is required to improve knowledge about predictive breastfeeding factors. Thus, the aim of this study was to describe the prevalence of CS and evaluate its effect on breastfeeding initiation among mothers of children under the age of two years in Abu Dhabi.

## 2. Materials and Methods 

### 2.1. Participants and Data Collection

The sample for this study is based on secondary data from an original sample of clinical and non-clinical data obtained from mothers with at least one infant under the age of two years. Participants for the original study included UAE nationals and non-nationals in the Emirate of Abu Dhabi, which represents 87% of the geographical landmass of the UAE [36]. All data were collected between March and September 2017. The subjects were randomly selected from the community and seven out of a total of 11 maternal and child health centers serving children in Abu Dhabi Capital City (two rural, one suburban, four urban), which provided the possibility of data collection across various areas of Abu Dhabi. Among the 1578 mothers from the clinics who were invited to participate, 1555 mothers agreed and were included in the study. Another 267 mothers from the community also agreed to participate in the study, resulting in a total sample size of 1822 mothers. The geographical distribution of the sample was 54%, 10.8%, and 35.2% of mothers recruited from rural areas, suburban areas, and urban areas respectively. 

Mothers with young children attending the centers were approached by the trained bilingual (Arabic and English) female research assistants, who provided oral and written information about the study. Consenting mothers who met the inclusion criteria of having at least one child under two years of age were interviewed by the research assistants using a structured questionnaire.

### 2.2. Study Instrument

A pre-tested questionnaire included family demographics (e.g., education, age, nationality, occupation), child’s information (e.g., birth weight and height, delivery mode), and infant feeding practices (e.g., initiation of breastfeeding, and rooming-in). Details of the methodology of the primary data have been previously described [23].

### 2.3. Study Inclusion and Exclusion Criteria

From the 1822 mother–child pairs, there were 1624 with complete data on all the variables of interest that were included in the analysis. 

### 2.4. Statistical Analysis

Data analysis was conducted using Statistical Package for the Social Science (IBM SPSS Statistics for Windows, Version 20.0. Armonk, NY: IBM Corp.). Both descriptive and inferential statistics were used to analyze the data. T-tests and chi-square tests were applied to analyze continuous and categorical data, respectively. Variables with significant *p*-value (<0.05) in univariate analysis were entered in multivariable logistic analysis with mode of delivery (vaginal delivery was coded as 0 and CS was coded as 1) as the dependent variable. In addition, body mass index (BMI) was categorized to underweight, normal, overweight, and obese in order to establish which category was associated with CS. Other variables such as sociodemographic characteristic (e.g., age, parent education, occupation, child gender, etc.) were considered as the independent variables. Odds ratio (OR) and 95% confidence intervals (CIs) were calculated with a significance level of *p*-value < 0.05. Furthermore, multinomial logistic regression analysis was used to investigate the impact of each mode of delivery (vaginal delivery, planned and emergency CS) on the early initiation of breastfeeding.

### 2.5. Ethics

The original study from which this data was extracted was approved by the Research Ethics Committee at Zayed University UAE (ZU17_006_F). Additional clearance was obtained from the Abu Dhabi Health Services Company. Informed consent was obtained from all participants prior to any data collection. Several measures were taken to ensure privacy and confidentiality throughout the study period by excluding personal identifiers during data collection. 

### 2.6. Definitions

Early initiation of breastfeeding: when the infant initiated breastfeeding within one hour after birth [37].

Delayed initiation of breastfeeding: when the infant initiated breastfeeding within more than one hour after birth.

Rooming-in: the child stays with mother in the same room during hospital stay.

Exclusive breastfeeding: the infant being fed only breast milk without any other oral intake, except medications and vitamins, for the first six months of life; it was calculated based on the last 24 h.

Breastfeeding support: the support and encouragement from family (mother, husband, other relatives, and other non-relatives) on breastfeeding

Breastfeeding advice and or discussion: any received information, positive or negative things about breastfeeding before or after delivery

Gestational age: a measure of the age of a pregnancy that is taken from the beginning of the woman’s last menstrual period

Preterm birth: the birth of a baby at <37 weeks GA

Arab nationality: all Emirati mothers and other Arab ones

Non-Arab nationality: Asian mothers and other nationalities

Cesarean section: a surgical procedure in which incisions are made through a woman’s abdomen and uterus to deliver her baby

## 3. Results

**Figure 1:**
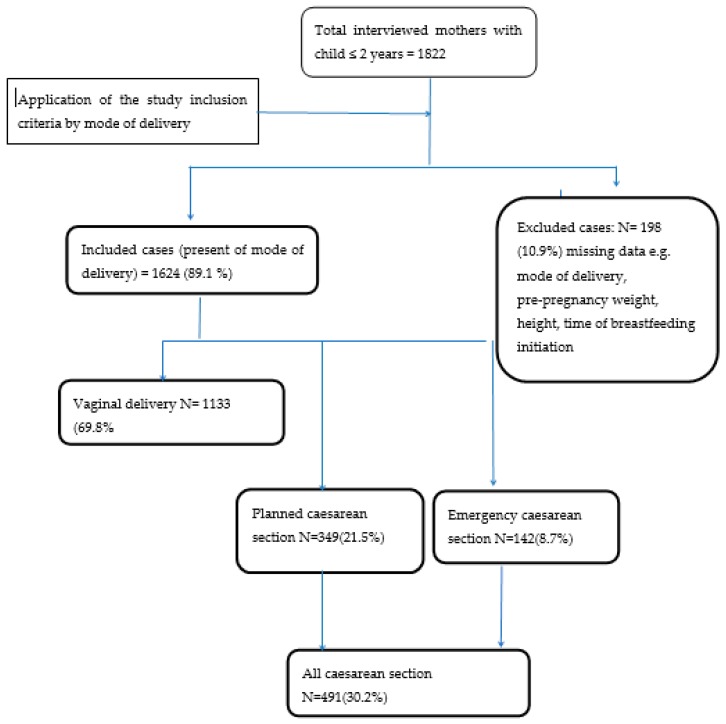
Study participants flow chart and main findings.

Secondary data analysis included 1624 samples from the original study, of which 491 (30.2%) participants reportedly delivered by CS, of which 349 (71.1%) delivered by planned CS, and 142 (28.9%) delivered by emergency CS (Figure 1).

The mean and standard deviation (SD) of the mothers’ age and children’s age were 30.1 (5.2) years and 8.1 months (5.9), respectively. The mean (SD) gestational age at delivery was 39.1 (1.9) weeks, and 6.5% of infants were preterm (GA <37 weeks). More than half (62.5%) of the mothers initiated early breastfeeding. Bivariate analysis showed that child age, birth weight, pre-pregnancy BMI, maternal education, initiation of breastfeeding, and rooming-in were associated with the mode of delivery (Table 1). Exclusive breastfeeding among infants between 0–6 months was 46.5% (332/710). 

By using logistic regression analysis, the different impacts of each mode of delivery (vaginal, planned CS, and emergency CS), and their impact on early initiation of breastfeeding, the study findings showed that vaginal delivery mothers were most likely to initiate early breastfeeding, followed by planned CS and emergency CS delivery (Table 3).

## 4. Discussion

One of the main findings was the high rate of CS (30.2%). This rate is more than double in comparison to the WHO set upper limit of 15% [3], and three times that of rates previously reported in the UAE (10% in 1995%) [1]. These data support the WHO reports that the rate of CS is increasing [2]. The results from this study exceed those for other Arab nations such as Saudi Arabia where a low rate of CS was reported (13.7%) [38] and Sudan (17.8%) [39]. Researchers attributed the varying trend of CS rate among nations to multiple factors including affluence [40], urbanization [40], and increases in preterm births [41]. For example, Boatin et al. [42] reported a low CS rate in South Sudan of 0.6% in comparison to the Dominican Republic, which reported 58.9%. CS rates also vary between public and private hospitals [43,44], which may be attributed to access to care.

This study indicated that non-Arab mothers were 1.3 times more likely to deliver by CS compared to Arab mothers. In multinational countries such as the UAE, the interpretation of such differences requires additional research. It should be noted that maternal age among non-Arab mothers was higher than Arab mothers, which may have contributed to the increased rates of CS in this group.

Advanced GA was found to be a protective factor for CS, (AOR 0.80, 95% CI = 0.75, 0.86) i.e., full-term babies were less likely to have been delivered by CS in comparison to preterm babies, which is a finding that is supported by previous research [41,45]. In addition, advanced maternal age was found to be associated with preterm birth [46,47,48]. Thus, we can conclude that factors such as advanced maternal age, preterm birth, and nationality are correlated. 

In contrast to our results, previous studies have found birth weight [43,49] and maternal education [40,49] to be associated with mode of delivery. Differences in the scope and approach to data collection may limit the ability to adequately compare current results with previous studies and warrant additional research. 

As with previous findings [50,51,52], the current study found an increased risk of CS in obese women. However, overweight was not associated with CS, and the findings of the current study were confined to the obese mothers. Regardless of the association between obesity and CS, it is well documented that maternal obesity is associated with a decreased intention and initiation of breastfeeding, a shortened duration of breastfeeding, a less adequate milk supply, and a delayed onset of lactogenesis II, and can thus be considered as a risk factor for adverse breastfeeding outcomes [53,54,55].

In addition to the CS prevalence and its associated factors, including the breastfeeding ones, the study also documented the impact of each mode of delivery on breastfeeding initiation. In line with others, the present results documented CS as a key risk factor for the delayed initiation of breastfeeding [24,28,56,57]. The researchers attributed the delay to many reasons, including pain [28], delayed lactogenesis [56], and feeding the newborn with formula [28,57]. 

The current study found that women who had a vaginal delivery 4.57 (3.16, 6.61) versus planned CS delivery 1.64 (1.09, 2.46) were four times more likely to initiate breastfeeding early. This finding is in line with several studies that documented vaginal delivery to be associated with an early initiation of breastfeeding [26,28,29,30]. This finding supports previous research that suggests rooming-in as a factor for the early initiation of breastfeeding, and CS decreases that opportunity. 

However, controversy exists between planned CS and emergency CS. Some researchers propose that planned CS is associated with a delayed initiation of breastfeeding [34] while others report that emergency CS is associated with a delayed initiation of breastfeeding [35], similar to our findings. The delayed initiation of breastfeeding after emergency CS could be attributed to the stress accompanying the labor and delivery, which is associated with a delayed onset of lactation [35]. 

Our study is the first of this kind in the UAE, using clinical and non-clinical data to describe CS and the initiation of breastfeeding. Several limitations should be noted. First, maternal indications and/or fetal indications of CS such as fetal distress, the type of anesthesia (general or regional) used during CS, and maternal and child condition such as morbidity and history of hospitalization after delivery by CS were not reported. These issues can affect the mode of delivery and its effect on breastfeeding practices. Future studies are needed to better describe the factors associated with CS in UAE populations.

## 5. Conclusions

This study suggests that the prevalence of CS is high in Abu Dhabi, UAE and that CS is associated with advanced maternal age, GA, nationality, and obesity. CS, and emergency CS in particular, was the main risk factor for a delayed initiation of breastfeeding. The study provides valuable information to aid with the development of appropriate strategies to reduce the CS rate in the UAE. Maternal literacy on CS choices, the importance of breastfeeding for child health, and additional guidance so that mothers and their families can achieve better breastfeeding outcomes are recommended to ensure successful breastfeeding practices.

## Figures and Tables

**Table 1:** Bivariate analysis of factors associated with caesarean section among mothers with children two years old and younger in Abu Dhabi, United Arab Emirates (UAE).

Variables			Mode of Delivery				
	Total (*n* = 1624)		Vaginal (*n* = 1133)		CS (*n* = 491)		
	Mean (SD), range		Mean (SD)		Mean (SD)		*p*-Value
Maternal age, years	30.1(5.2)		29.9(5.3)		31.0(4.8)		<0.001
Child age, months (median 6, interquartile range 9)	8.1(5.9)		8.3(6.0)		7.6(5.7)		0.042
Gestational age, weeks	39.1(1.9)		39.4(1.7)		38.6(2.1)		<0.001
Birth weight, grams	3079(518)		3110(463)		3008(621)		<0.001
Mother pre-pregnancy BMI	23.9(3.8), (15.2, 64.9)		23.7(3.7)		24.3(4.2)		0.002
	**N**	**%**	**N**	**%**	**N**	**%**	
Child Gender							
Male Female	799	49.2	560	49.4	239	48.7	0.781
	825	50.8	573	50.6	252	51.3	
Nationality by category							
Arab Non-Arab	1056	65.0	757	66.7	299	60.9	0.049
	568	35.0	376	33.3	192	39.1	
Marital status							
Married Unmarried	1602	98.6	1116	98.5	486	99.0	0.440
	22	1.4	17	1.5	5	1.0	
Initiation of breastfeeding							
Delayed initiated Early initiated	609	37.5	329	29.0	280	57.0	< 0.001
	1015	62.5	804	71.0	211	43.0	
Rooming-in							
Yes No	1562	96.2	1101	97.2	461	93.9	0.002
	62	3.8	32	2.8	30	6.1	
Mother’s education							
<Secondary level ≥secondary level	65	4.0	53	4.7	12	2.4	0.035
	1559	96.0	1080	95.3	479	97.6	
Father’s education							
<Secondary level ≥secondary level	31	1.9	23	2.0	8	1.6	0.588
	1593	98.1	1110	98.0	483	98.4	
Mother occupation							
Housewives Employed	1008	62.1	695	61.3	313	63.7	0.533
	616	37.9	438	38.7	178	36.3	
Child order							
1st order >1st order	1038	63.9	399	35.2	187	38.1	0.269
	1038	63.9	304	61.9	304	61.9	

Multivariable logistic regression analysis indicated that several factors were positively associated with caesarean section (CS). These included: advanced maternal age (Adjusted Odd Ratio (AOR) = 1.04, 95% confidence interval (CI) = 1.02, 1.07), nationality (non-Arab) (AOR = 1.30, 95% CI = 1.02, 1.65), and body mass index (BMI) status (obesity) (AOR = 1.79, 95% CI = 1.15, 2.79). Gestational age (GA); however, (AOR = 0.80, 95% CI = 0.75, 0.86) was found to be negatively associated with CS (Table 2).

**Table 2:** Multivariable logistic regression analyses of factors associated with caesarean section among mothers with children two years old and younger in Abu Dhabi, UAE.

Variable		Adjusted Odds Ratio	95% Confidence Interval	*p*-Value
Advanced maternal age, years		1.04	1.02, 1.07	< 0.001
Gestational age, weeks		0.80	0.75, 0.86	<0.001
Child’s birth weight, grams		1.00	1.00, 1.00	0.283
Nationality	Non-Arab	1.36	1.08, 1.71	0.009
	Arab	Reference		
Maternal education	≥Secondary	1.88	0.94, 3.75	0.072
	<Secondary	Reference		
Mother pre-pregnancy BMI	Underweight (<18.5)	1.12	0.56, 2.26	0.746
	Normal (18.5–24.9)	Reference		
	Overweight (25–29.9)	1.10	0.85, 1.42	0.477
	Obese (≥30)	1.79	1.15, 2.79	0.010

From the total (1624), 1133 (69.8%), 349 (21.5%), and 142 (8.7%) delivered vaginally, by planned CS and by emergency CS, respectively. Among mothers who initiated breastfeeding early (1015), the rates of an early initiation of breastfeeding were 804 (79.2%) 95% CI (76.4, 82.0), 162 (16.0%) 95% CI (10.4, 21.6), and 49 (4.8%) 95% CI (1.2, 10.8) for vaginal deliveries, planned CS, and emergency CS, respectively.

**Table 3:** Impact of mode of delivery (vaginal delivery, planned CS, and emergency CS) in comparison to each other on an early initiation of breastfeeding using logistic regression analysis.

Mode of Delivery	95% Confidence Interval (CI)	*p*-Value	AOR ^1^ (95% CI)	*p*-Value	AOR (95% CI)	*p*-Value
Vaginal delivery	4.5 (3.16, 6.61)	< 0.001	2.78 (2.17, 3.56)	< 0.001	Reference	
Planned CS	1.64 (1.09, 2.46)	0.017	Reference		0.36 (0.28, 046)	< 0.001
Emergency CS	Reference		0.61 (0.41, 0.91)	0.017	0.22 (0.15, 0.32)	< 0.001

^1^ AOR: Adjusted for advance maternal age, gestational age, and mother’s BMI.
